# Nitrobenzene induced methemoglobinemia treated with manual exchange transfusion in a resource-limited setting: a case report

**DOI:** 10.1097/MS9.0000000000003147

**Published:** 2025-03-27

**Authors:** Prabhat Silwal, Sanjan K. Sah, Rojeena Adhikari, Sophiya Maharjan, Nirish Vaidya, Samjhana Basnet, Ajay Bhatt

**Affiliations:** a Dhulikhel Hospital, Kathmandu University School of Medical Sciences, Dhulikhel, Kavrepalanchowk, Nepal; b Department of Internal Medicine, Dhulikhel Hospital, Kathmandu University School of Medical Sciences, Dhulikhel, Kavrepalanchowk, Nepal; c Department of General Practice and Emergency Medicine, Dhulikhel Hospital, Kathmandu University School of Medical Sciences, Dhulikhel, Kavrepalanchowk, Nepal; d Department of Emergency Medicine, John A. Burns School of Medicine, University of Hawaii, Honolulu, Hawaii, USA

**Keywords:** exchange transfusion, methemoglobinemia, nitrobenzene, poisoning, resource-limited setting

## Abstract

**Introduction and importance:**

Nitrobenzene poisoning can result in life-threatening methemoglobinemia. Therefore, immediate management is critical.

**Case presentation:**

We report the case of a 45-year-old female, who was brought to our emergency department three hours after ingesting approximately 50 mL of a 35% solution of nitrobenzene. She displayed decreased consciousness, cyanosis, low peripheral oxygen saturation (SpO2) and a saturation gap of 21.9%. Despite high-flow oxygen supplementation, cyanosis persisted, and SpO2 remained low. Nitrobenzene poisoning with methemoglobinemia was diagnosed on the basis of her clinical features. She was treated with manual therapeutic whole blood exchange (TWBE), intravenous vitamin C, and supportive care. The patient underwent manual exchange transfusion of 2365 mL of whole blood (60% of her estimated total blood volume) over 5 days. After treatment, the patient recovered and was discharged from the hospital.

**Clinical discussion:**

The patient was diagnosed on the basis of clinical features because tests to measure the level of methemoglobin were unavailable. Methylene blue, which is first-line treatment for methemoglobinemia, was not available. Therefore, the patient was treated with manual TWBE and intravenous vitamin C. Several published reports have illustrated the successful use of TWBE in the treatment of methemoglobinemia in settings where methylene blue was unavailable.

**Conclusion:**

A thorough clinical evaluation is key in diagnosing methemoglobinemia in circumstances where specific tests to detect methemoglobin are not available. Treatment with methylene blue can be ineffective, contraindicated or unavailable in some circumstances. In such situations, alternative therapies such as TWBE and intravenous vitamin C should be considered.

## Introduction

Nitrobenzene is a synthetic, aromatic compound that exists as a colorless to pale yellow colored oily liquid with a smell similar to that of bitter almonds^[1]^. It is used as an ingredient in inks, dyes, polishes and disinfectants. It is also used in the manufacture and processing of petroleum, aniline, plastics, pesticides and drugs such as acetaminophen. Inhalational, dermal or oral exposure to nitrobenzene can be toxic to humans. The main toxic effect is methemoglobinemia. Other reported effects include toxicity to the liver, spleen, kidney and nervous system resulting in coma and even death^[1]^.

**HIGHLIGHTS**

- Nitrobenzene poisoning is uncommon but can lead to significant toxicity in humans.
- In this unique case, the diagnosis was made on the basis of clinical features as confirmatory tests were not available.
- Furthermore, an atypical approach to treatment was adopted owing to the unavailability of the first-line drug in our setting.
- This case demonstrates the importance of thorough clinical evaluation and appropriate clinical judgment for the diagnosis and treatment.

Methemoglobinemia is a rare disorder characterized by oxidation of hemoglobin to form methemoglobin^[2]^. More specifically, the iron moiety of hemoglobin is oxidized from the ferrous state (Fe^2+^) to the ferric state (Fe^3+^). Consequently, there is leftward shift of the oxygen dissociation curve. This reduces oxygen delivery to tissues and can lead to hypoxia. Methemoglobinemia can result either from an inherited genetic mutation, or following exposure to substances that cause oxidation of hemoglobin. Exposure to some aniline dyes, pesticides, nitrate compounds as well as medicines like benzocaine, dapsone, lidocaine, and sulfonamides can lead to acquired methemoglobinemia^[2,3]^. Dapsone (an antimicrobial and anti-inflammatory agent) and benzocaine (a local anesthetic) are commonly used medications that frequently cause methemoglobinemia^[3]^.

Here, we report a case of self-poisoning with a 35% solution of nitrobenzene in a 45-year-old female presenting with features consistent with severe methemoglobinemia. We discuss our approach to the diagnosis and treatment taking into account the exceptional challenges that we encountered owing to our limited resources. This case report has been prepared in accordance with the case report (CARE; Supplementary Digital Content, http://links.lww.com/MS9/A756) guidelines^[4]^ and written informed consent was obtained from the patient for publication of this case report.

## Case details

A 45-year-old female presented to our emergency department three hours after ingesting approximately 50 mL of a 35% solution of nitrobenzene with suicidal intent. She had also ingested homemade alcohol 2 hours prior to this incident. Furthermore, one episode of vomiting had been induced by feeding her soap water at home. Family members brought the labeled bottle of nitrobenzene to the hospital.

On examination, she had a Glasgow coma scale of 8/15 with eye sub-score of 2, motor sub-score of 3, and verbal sub-score of 3 (E2M3V3). Both central and peripheral cyanosis were present. Her heart rate was 109 beats per min, respiratory rate was 10 breaths per min and blood pressure was 100/60 mmHg. Her oxygen saturation in peripheral capillaries measured by pulse oximetry (SpO_2_) was 78% when she was under oxygen supplementation at 15 L/min through an oxygen mask with a reservoir bag. Cyanosis did not improve after oxygen supplementation. Other parameters of general and systemic examination were within the normal limits.

Based on the clinical features, a diagnosis of nitrobenzene induced methemoglobinemia was made. In the emergency room, the patient’s airway was cleared by suctioning excess secretions, endotracheal intubation was performed and mechanical ventilation with 100% oxygen was initiated. Intravenous resuscitation with normal saline was also started simultaneously. Decontamination was done by changing the patient’s clothes and washing her body surface. The patient was given intravenous vitamin C 500 mg in the emergency room. After initial stabilization in the Emergency Department, the patient was admitted in the intensive care unit (ICU). Her laboratory test parameters are detailed in Table 1 and arterial blood gas (ABG) analysis results are listed in Table 2. The saturation gap was 21.9%. Her chest radiograph and electrocardiogram were unremarkable.

**Table 1:** Laboratory parameters at presentation and various points during the hospital stay

Laboratory parameter	Level at presentation	Day 3	Day 5	Day 7	Laboratory reference range
Total leukocyte count	12 400/µL		11 300		4000–11 000/µL
**Differential leukocyte count**					
Neutrophils	82%		80%		50–70%
Lymphocyte	15%		14%		20–40%
Monocytes	03%		05%		2–8%
Eosinophils	00		00		2–8%
Basophils	00		00		0–1%
Hemoglobin	14.5 g/dL		10 g/dL		11–15 g/dL
Platelet count	197 000/µL		192 000/µL		150 000–450 000/µL
Prothrombin time (PT)	12 s		13 s		10–13 s
International normalized ratio(INR)	1.0		1.0		
Random plasma glucose	90 mg/dL				60–150 mg/dL
Plasma cholinesterase	4933 U/L				3930–10 800 U/L
CRP quantitative	6 mg/dL			28 mg/dL	<10 mg/L
Creatinine	0.4 mg/dL	0.3 mg/dL	0.2 mg/dL	0.3 mg/dL	0.5–1.1 mg/dL
Urea	22 mg/dL	7 mg/dL	10 mg/dL	11 mg/dL	10–45 mg/dL
Serum sodium	148 mEq/L	141 mEq/L	140 mEq/L	140 mEq/L	135–148 mEq/L
Serum potassium	3.9 mEq/L	3.5 mEq/L	4.1 mEq/L	4.2 mEq/L	3.5–5.3 mEq/L
Total bilirubin	0.4 mg/dL				0.3–1.4 mg/dL
Direct bilirubin	0.2 mg/dL				<0.3 mg/dL
Aspartate transaminase (AST)	758 IU/L		72 IU/L		<40 IU/L
Alanine transaminase (ALT)	289 IU/L		105 IU/L		<40 IU/L
Alkaline phosphatase (ALP)	76 IU/L				42–128 IU/L
Gamma glutamyl transferase (GGT)	164 U/L				<45 U/L

**Table 2:** Arterial blood gas (ABG) analysis parameters

Arterial blood gas (ABG) analysis parameter	Level at presentation	Day 1	Day 3	Day 5	Day 7	Laboratory reference range
pH	7.31	7.32	7.47	7.43	7.39	7.35–7.45
Partial pressure of carbon dioxide (pCO_2_)	40.3 mmHg	46.9 mmHg	40.6 mmHg	38.3 mmHg	41 mmHg	35–45 mmHg
Partial pressure of Oxygen (pO_2_)	303 mmHg	290 mmHg	102 mmHg	137 mmHg	86 mmHg	90–110 mmHg
Oxygen saturation (SaO_2_)	99.9%	99.9%	98.2%	99.2%	96.5%	
Fraction of inspired oxygen (FiO_2_)	100%	100%	50%	50%	40%	
Bicarbonate	19.9 mmol/L	22.7 mmol/L	29 mmol/L	26.1 mmol/L	24.3 mmol/L	22–26 mmol/L
Anion gap	23.7 mEq/L	18.8mEq/L	14.2mEq/L	14.7mEq/L	14.3mEq/L	8–12 mEq/L

Despite our best efforts, we could not procure methylene blue for the treatment of methemoglobinemia due to resource limitations. Hence, manual therapeutic whole blood exchange (TWBE) was initiated on day 1 of her admission. Based on the patient’s weight of 60 kg and an estimated blood volume of 65 mL/kg, we estimated the total blood volume of the patient to be 3900 mL. Simultaneous transfusion of one pint of whole blood and phlebotomy of one pint of blood was done on each day for 5 consecutive days. Hence, a total of 2365 mL (60% of estimated total blood volume) of whole blood was exchanged. TWBE was stopped after clinical improvement in terms of resolution of cyanosis and metabolic acidosis as well as decreased oxygen requirement. No complications were encountered during TWBE. The patient was also treated with intravenous vitamin C 500 mg three times a day, intravenous multivitamin supplementation, intravenous pantoprazole 40 mg once a day, intravenous fluids, input/output charting and continuous monitoring of vital signs. The ventilatory support required by the patient progressively decreased and the patient was extubated on day 6 of her admission.

Subsequently, her clinical condition improved and she was transferred to the medicine ward on day 8. She was discharged on the 9th day after admission following psychiatry consultation and psychosocial counseling in view of attempted suicide. After discharge, she was advised to take tablets 500 mg of vitamin C two times a day for two more weeks. The patient was advised to follow up after 1 week. However, she did not visit the hospital for a follow-up visit. The patient was well and did not report any medical complaints on a telephone interview 1 year after discharge.

## Discussion

Nitrobenzene poisoning is uncommon in Nepal when compared to poisoning with other substances like insecticides and drugs^[5]^. A search in the PubMed database yielded only four cases of nitrobenzene poisoning reported in Nepal^[6-9]^. However, when it does occur, it can lead to lethal toxicity^[1]^.

The major toxic effect of nitrobenzene is methemoglobinemia which can then lead to tissue hypoxia^[1]^. In an otherwise healthy person with methemoglobinemia, clinical presentation varies according to the methemoglobin level^[2,3]^. The person may be asymptomatic if the methemoglobin level is less than 10% of the total hemoglobin. Cyanosis can be seen at levels more than 10%. Neurologic symptoms such as anxiety, headache and dizziness can occur at levels more than 20%. Levels more than 30% can lead to fatigue, confusion and tachypnea. A methemoglobin level of more than 50% can result in arrhythmias, acidosis, seizures, coma, and death^[2,3]^. Other unique clinical features include cyanosis refractory to oxygen therapy, low SpO_2_ unresponsive to oxygen supplementation and a saturation gap of more than 5%^[2,3]^. Saturation gap is the difference between the values of oxygen saturation measured by ABG analysis (SaO_2_) and pulse oximetry (SpO_2_)^[3]^.

Definitive diagnosis of methemoglobinemia and determination of methemoglobin levels is possible by pulse co-oximetry or the Evelyn-Malloy test^[2,3]^. However, these tests were not available at our center. Hence, our diagnosis of methemoglobinemia was based on the history of ingestion of nitrobenzene, cyanosis refractory to oxygen therapy, low SpO2 unresponsive to oxygen supplementation and a saturation gap of 21.9%. We estimated our patient’s methemoglobin level to be more than 50% due to the patient’s clinical presentation of decreased consciousness and metabolic acidosis^[2]^.

Treatment of acquired methemoglobinemia includes decontamination and cutting off further exposure to the causative agent, non-specific supportive therapy and specific therapies to reduce the level of methemoglobin^[2]^. Specific therapy is recommended when the methemoglobin level is more than 30% in asymptomatic patients and more than 20% in symptomatic patients^[2,10]^.

The first-line specific therapy for methemoglobinemia is intravenous methylene blue which via oxidation of nicotinamide adenine dinucleotide phosphate (NADPH) reduces methemoglobin to hemoglobin^[2]^. Adjunctive therapy with vitamin C is recommended as it reduces excessive oxidative stress. Although vitamin C can directly reduce methemoglobin back to hemoglobin, this is a slow process. In cases of refractory methemoglobinemia, TWBE or hyperbaric oxygen therapy should be considered^[2]^.

Methylene blue is contraindicated in conditions such as glucose-6-phosphate dehydrogenase deficiency (G6PD) and hemolytic anemia^[2,11]^. In such circumstances, alternative therapies like vitamin C and TWBE should be considered^[2]^.

TWBE results in the removal of the toxins in the plasma, hemolyzed plasma and the methemoglobin in the red blood cells of the patient^[11]^. These removed blood components are replaced by transfusion of whole blood which can deliver more oxygen to the tissues. TWBE has been used successfully for the treatment of methemoglobinemia in situations where methylene blue was unavailable^[11]^. At our center, another case of methemoglobinemia had also been treated with TWBE due to the unavailability of methylene blue^[9]^.

The decrease in hemoglobin seen in our patient could have been caused by hemolysis which is frequently associated with methemoglobinemia resulting from exposure to oxidizing substances^[10]^.

One limitation of this case report is the absence of follow-up patient visits. We were unable to study the changes in the hemoglobin level following discharge. Moreover, this is a report of a single case that demonstrated the usefulness of clinical methods in diagnosis as well as the role of alternative therapies in treatment of methemoglobinemia. Further studies to assess the role of these clinical methods and alternative therapies are warranted.

## Conclusions

Methemoglobinemia can be difficult to diagnose in health-care settings without tests that directly detect methemoglobin in blood. A thorough clinical history and detailed physical examination can help to reach a diagnosis in such circumstances. Alternative therapies for methemoglobinemia such as TWBE and intravenous vitamin C can be life-saving in settings where treatment with methylene blue is ineffective, contraindicated or unavailable.

## Data Availability

All data that support the findings of this study are included in this article. Further inquiries can be directed to the corresponding author.
